# Potent Virucidal Activity In Vitro of Photodynamic Therapy with *Hypericum* Extract as Photosensitizer and White Light against Human Coronavirus HCoV-229E

**DOI:** 10.3390/pharmaceutics14112364

**Published:** 2022-11-02

**Authors:** Beatriz Praena, Marta Mascaraque, Sabina Andreu, Raquel Bello-Morales, Edgar Abarca-Lachen, Valentina Rapozzi, Yolanda Gilaberte, Salvador González, José Antonio López-Guerrero, Ángeles Juarranz

**Affiliations:** 1 Departamento de Biología Molecular, Universidad Autónoma de Madrid, Edificio de Biología, Darwin 2, Cantoblanco, 28049 Madrid, Spain; 2 Departamento de Biología, Universidad Autónoma de Madrid, Edificio de Biología, Darwin 2, Cantoblanco, 28049 Madrid, Spain; 3 Instituto Ramón y Cajal de Investigaciones Sanitarias, IRYCIS, 28040 Madrid, Spain; 4 Centro de Biología Molecular Severo Ochoa, CSIC-UAM, Cantoblanco, 28049 Madrid, Spain; 5 Facultad de Ciencias de la Salud, Universidad San Jorge, 50830 Villanueva de Gállego, Spain; 6 Department of Medicine, University of Udine, 33100 Udine, Italy; 7 Hospital Miguel Servet, Servicio de Dermatología, 50009 Zaragoza, Spain; 8 Departamento de Medicina y Especialidades Médicas, Universidad de Alcalá, 28805 Madrid, Spain

**Keywords:** HCoV-229E, virucide, coronavirus, *Hypericum* extract, photosensitization

## Abstract

The emergent human coronavirus SARS-CoV-2 and its high infectivity rate has highlighted the strong need for new virucidal treatments. In this sense, the use of photodynamic therapy (PDT) with white light, to take advantage of the sunlight, is a potent strategy for decreasing the virulence and pathogenicity of the virus. Here, we report the virucidal effect of PDT based on *Hypericum* extract (HE) in combination with white light, which exhibits an inhibitory activity of the human coronavirus HCoV-229E on hepatocarcinoma Huh-7 cells. Moreover, despite continuous exposure to white light, HE has long durability, being able to maintain the prevention of viral infection. Given its potent in vitro virucidal capacity, we propose HE in combination with white light as a promising candidate to fight against SARS-CoV-2 as a virucidal compound.

## 1. Introduction

Coronaviruses (CoVs) are enveloped positive-sense single-stranded RNA viruses that cause respiratory, gastrointestinal, hepatic and neurological problems in both humans and animals [1]. Human CoVs (HCoVs) HCoV-NL63, HCoV-229E, HCoV-OC43 and HCoV-HKU have been described as responsible for respiratory tract infections and common colds [2,3]. However, SARS-CoV and MERS-CoV, which cause Severe Acute Respiratory Syndrome (SARS) and Middle East Respiratory Syndrome (MERS), respectively, have received worldwide attention in recent decades due to their ability to cause outbreaks of serious infections [4]. Recently, the appearance of a new coronavirus, SARS-CoV-2, the etiological agent of COVID-19, led the WHO to declare this public health emergency a pandemic in March 2020. The COVID-19 pandemic is responsible, at the time of writing this paper, for more than 450 million confirmed cases and more than 6 million deaths worldwide [5].

The U.S. Food and Drug Administration (FDA) has authorized various antiviral agents and monoclonal antibodies for the treatment of SARS-CoV-2, which include remdesivir, paxlovid, molnupiravir, dexamethasone, convalescent plasma-containing neutralizing antibodies and bebtelovimab [6]. Nonetheless, these strategies have been authorized for emergency use only and a concrete and totally effective treatment is still far from being discovered [7,8,9]. The virucidal treatments are also crucial to prevent or reduce rates of viral infections and the lack of effective drugs to play this role against COVID-19 makes the prompt search for useful virucidal drugs imperative. In this sense, the use of photodynamic therapy (PDT) can be a valid strategy [10]. PDT is a clinically approved technique, mainly applied in treating cancerous and non-cancerous diseases. PDT is minimally invasive, and it is based on the use of three elements: a photosensitizer (PS) compound, a light of appropriate wavelength and oxygen [11]. Each of these components is not toxic per se but their combination induces a photochemical reaction that leads to the formation of reactive oxygen species (ROS), which induce a lethal oxidative effect [12,13]. PDT can be applied not only in cancerous lesions, but also in the treatment of infectious diseases caused by several microorganisms, which include bacteria [14,15], fungi [16] and viruses [17]. PDTs used for this purpose are called antimicrobial PDTs (aPDTs). The most frequent PSs are the pheno-thiazines toluidine blue (TBO) and methylene blue (MB), as well as the precursors of the protoporphyrin IX (PpIX), aminolevulinic acid (ALA) and its derivate, methyl-aminolevulinate (MAL) [18]. Regarding viruses, aPDTs have been frequently used in the treatment of human papillomavirus (HPV), human immunodeficiency virus (HIV) and herpes simplex virus type 1 (HSV-1) [10,19,20].

Some studies have shown how these therapies may be successful as pre- or post-treatment against SARS-CoV-2 [21,22]. Treatment with MB exposed to red light led to several lesions in RNA viruses [23], causing damage to the genome and chemical modifications. Other in vitro studies and clinical trials corroborated the synergistic effect of this aPDT in combination with riboflavin [24] or radachlorin [25], since the antiviral activity of MB or radachlorin against SARS-CoV-2 showed an increase when viral particles were irradiated with white light [25].

In this context, *Hypericum* extracts (HE) from the aerial parts of *Hypericum perforatum L*. (perennial herbaceous plant), also known as St. John’s wort, have been used to treat various medical conditions, including microbial infections. The bioactive metabolites of HE have been reported with naphthodianthones (hypericin and pseudohypericin), phloroglucinols (hyperforin and adhyperforin) and flavonoids [26,27]. It has been stated that the most light-dependent components are naphthodianthones (hypericin and pseudohypericin), while the effect of phloroglucinols is independent of light [26,28,29]. Hypericin shows several absorption peaks in the visible spectrum (e.g., maximum absorbance at 550 and 588 nm in ethanol) and fluorescence emission at approximately 600 nm in ethanol [30]. This compound is a very promising agent for PDT in the oncology, diagnosis and therapy of bladder tumor cells, nasopharyngeal cancer, nonmelanoma skin cancer, or cutaneous lymphoma, among other diseases [31]. Regarding the antimicrobial activity of hypericin, several extracts have been shown to have an inhibitory effect against Gram-positive and Gram-negative bacteria and even against *Candida albicans* with and without light activation [32]. The virucidal activity of this compound has also been studied against enveloped viruses including HSV-1, HIV and cytomegalovirus (CMV) [10,19].

Here we report the virucidal effect of aPDT based on *Hypericum* extract (HE) in combination with white light, which exhibits an inhibitory activity of the human coronavirus HCoV-229E on hepatocarcinoma Huh-7 cells. In addition, despite continuous exposure to white light, HE has long durability, able to maintain the prevention of viral infection. Given its potent in vitro virucidal capacity, we propose HE in combination with white light as a promising candidate to fight against coronaviruses as a virucidal compound.

## 2. Materials and Methods

### 2.1. Cell Cultures

The Huh-7 hepatocarcinoma cell line was generously provided by Dr. Sonia Zúñiga (CNB-Spanish National Centre for Biotechnology, Madrid, Spain). This cell line was obtained from a liver tumor in a 57-year-old Japanese male and was used to test HCoV-229E infection [33]. The Vero cell line (ATCC-CCL81), which was derived from the kidney of an adult African green monkey (Cercopithecus aethiops) was purchased from the American Type Culture Collection (ATCC, Manassas, VA, USA). All cell lines were cultured in growth medium (GM) containing low-glucose Dulbecco’s modified Eagle medium (Life Technologies, Paisley, UK) supplemented with 5% fetal bovine serum (FBS), 1% (*v*/*v*) penicillin G (50 U/mL) and streptomycin (50 µg/mL) (HyClone Laboratories, Logan, UT, USA). Cells were maintained at 37 °C, in a humidified atmosphere of 5% CO_2_ (Heraeus HERAcell, Thermofisher. Waltham, MA, USA).

### 2.2. Viruses

HCoV-229E-expressing green fluorescent protein (GFP) was generously provided by Dr. Volker Thiel (University of Bern, Bern, Switzerland). This virus was propagated on Huh-7 cells and the viral titer of the stocks was calculated by an endpoint dilution assay. Briefly, sub-confluent monolayers of Huh-7 cells were plated in 96-well tissue culture dishes and cultured in GM. Serial dilutions (10-1 to 10-9) of HCoV-229E were inoculated onto replicate cell cultures. Cells were then incubated at 33 °C in a humidified atmosphere containing 5% CO_2_ for 5 days. Finally, the 50% tissue culture infectious dose (TCID50) per mL was determined, considering the final dilution that showed cytopathic effect (CPE) and calculated using the Reed and Muench method [34]. Fluorescence herpes simplex virus type 1 (HSV-1) named K26-GFP was obtained by fusing GFP to the HSV-1 capsid protein VP26 [26]. HSV-1 K26-GFP was propagated and titrated by an endpoint dilution assay on Vero cells and used to obtain comparative results with HCoV-229E.

### 2.3. Compounds

The *Hypericum* extract (HE) (Farmaquímica Sur, Málaga, Spain), is a commercial controlled hydrophilic extract obtained as a lyophilized powder of *Hypericum perforatum*. The extraction solvent was water-ethanol (8:1), with less than 10% of maltodextrin as an excipient. The extract had 0.35% hypericin according to the UV titer. Further product details are shown in the attached data sheet provided by the suppliers in the supplementary material. A stock solution was prepared at 0.30% (*w*/*v*) in water, and subsequently s tested at 1 μg/mL and 2 μg/mL, in phenol red-free DMEM supplemented with 2% (*v*/*v*) FBS. Maury’s article indicated that 2 µg/mL HE did not induce toxicity in the absence of light, whereas higher concentrations did, and so this concentration was selected [26]. Methylene blue (MB) (Merck Chemicals, Darmstadt, Germany) was used as a positive control (0.25 μg/mL in DMEM). Stock solutions were prepared in distilled water at a concentration of 10 mg/mL. The stocks were then diluted in phenol red-free DMEM supplemented with 2% (*v*/*v*) FBS to the desired final concentrations.

### 2.4. Cell Viability Assay

The cytotoxic effects of aPDT (MB/HE plus white light) on Huh-7 and Vero cells were analyzed by a colorimetric MTT assay (Roche Cell Proliferation Kit I, Roche, Basel, Switzerland). When the cultured cells reached an approximate 60–70% confluence, they were subjected to three different treatments: (a) incubation with the PS alone for 1 h–MB (0.25 μg/mL) or HE (1–2 μg/mL) [26]; (b) irradiation at 13.2 J/cm^2^ with white light (420–700 nm, 90 mW/cm^2^); (c) subjection to the aPDT (MB/HE plus white light) under the same conditions as in the treatment with the PS alone. After 24 h of different treatments, cells were incubated with a final concentration of 0.5 mg/mL of MTT solution in a humidified atmosphere for 4 h at 37 °C. The resulting formazan precipitate was dissolved with 10% SDS in 0.01 M of HCl and absorbance was measured at 595 nm using a plate reader (SpectraFluor, Tecan, Männedorf, Switzerland). The readouts obtained from the MTT assay were further normalized to the control value of non-irradiated and non-treated cells, where the viability value was set to 100%.

### 2.5. Treatments and Infections

HCoV-229E and HSV1 K26-GFP were mixed with either HE (1 or 2 μg/mL) or MB (0.25 μg/mL) for 1 h in 100 µL of red-free DMEM supplemented with 2% FBS in dark. During the treatment, the compounds were irradiated or not with a white light source (420–700 nm, 90 mW/cm^2^) at a dose of 13.2 J/cm^2^, 15 min before the treatment time was over. The light source was placed under the culture plates so that viruses were irradiated directly from below, avoiding any possible shielding effects exerted by the culture medium or the treatments. The temperature reached in the vial after light exposure did not exceed 36 °C. Then, Huh-7 and Vero cells cultured at 80% of confluence in 96-well plates were infected with the treated or mock-treated mixture at a multiplicity of infection (m.o.i) of 0.5 or 1 with HCoV-229E or HSV-1 K26-GFP, respectively (Figure S1), for 1 h. Subsequently, the infected cells were rinsed twice with PBS and maintained in DMEM with 10% FBS at 35 °C, in a humidified atmosphere of 5% CO_2_. The infectivity was determined by qualitative methods, such as CPE and observation of virus-GFP signal using the Zeiss Axiovert 200 inverted microscope (Carl Zeiss, Oberkochen, Germany).

### 2.6. Photosensitizers-Degradation

The photo-degradation of HE and MB was evaluated according to the infection rate. For this, PSs alone (2 µg/mL HE, 0.25 µg/mL MB) were pre-irradiated for 1 or 3 h with white light (420–700 nm, 90 mW/cm^2^) in phenol red-free DMEM supplemented with 2% FBS. After that, HCoV-229E was mixed with each PS for 1 h in the dark, and 15 min before the end of the treatment, the PSs with the viruses were exposed to 13.2 J/cm^2^ of white light. Subsequently, Huh-7 cells at 80% of confluence were infected with the treatment mixture at an m.o.i of 0.5 with HCoV-229E. The effectiveness of PSs was evaluated 48 h after the infection by virus titration (Figure S2).

### 2.7. Flow Cytometry

For flow cytometry analysis, HCoV-229E was treated as described in Section 2.5. At 48 h post-infection (h p.i), cells were dissociated by incubation with 0.05% trypsin/0.1% EDTA (Invitrogen, Carlsbad, CA, USA) at room temperature, washed and fixed in 4% paraformaldehyde for 15 min. Subsequently, cells were rinsed and resuspended in PBS. The analysis of the viral GFP signal was performed using a FACSCalibur Flow Cytometer (BD Biosciences, San Jose, CA, USA).

### 2.8. Statistics

At least three biological replicates were performed for each assay. A Mann–Whitney U-test for independent measures was performed to compare the mean values of each data set, and *p*-values < 0.05 were classified as statistically significant (using Prism software v8.0.1, GraphPad Software, Inc., San Diego, CA, USA).

## 3. Results

### 3.1. Effect of aPDT in Vero and Huh-7 Cells

**Figure 1:**
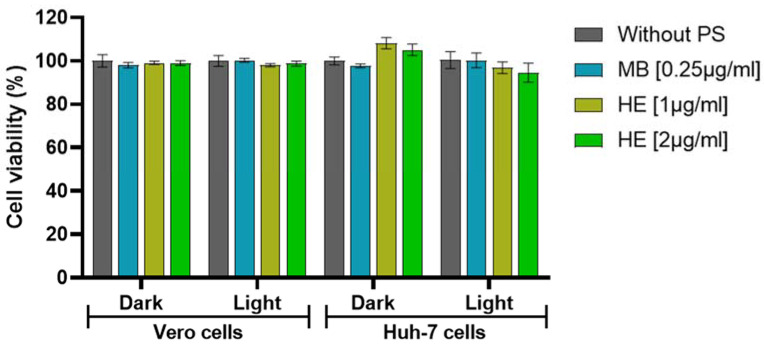
Effects on cell viability in Vero or Huh-7 cells induced by incubation with MB and HE with and without subjection to white light irradiation (420–700 nm, 90 mW/cm^2^) at a dose of 13.2 J/cm^2^. Cell toxicity was evaluated by MTT assay and calculated as the percentage of viability compared to untreated cells; columns represent the mean viability (*n* = 3) ± S.D. after exposure to the compounds.

Before evaluating the virucidal properties of HE, its effects on cell viability with and without subjection to white light irradiation (420–700 nm, 90 mW/cm^2^) were assessed by an MTT assay in Vero and Huh7 cells. As a positive control, the well-known MB photosensitizer was used. Figure 1 shows the effects on cell viability induced by HE at two different concentrations (1 and 2 µg/mL) and MB with and without subjection to white light irradiation (420–700 nm, 90 mW/cm^2^) at a dose of 13.2 J/cm^2^, as well as the effects on cell viability of the irradiation with white light alone in Vero and Huh-7 cells. None of the treatments reduced cell survival by more than 5%. Therefore, treatment with aPDT showed no effect on cell viability.

### 3.2. Effect of Irradiated and Non-Irradiated HE and MB against HSV-1 Infection

**Figure 2:**
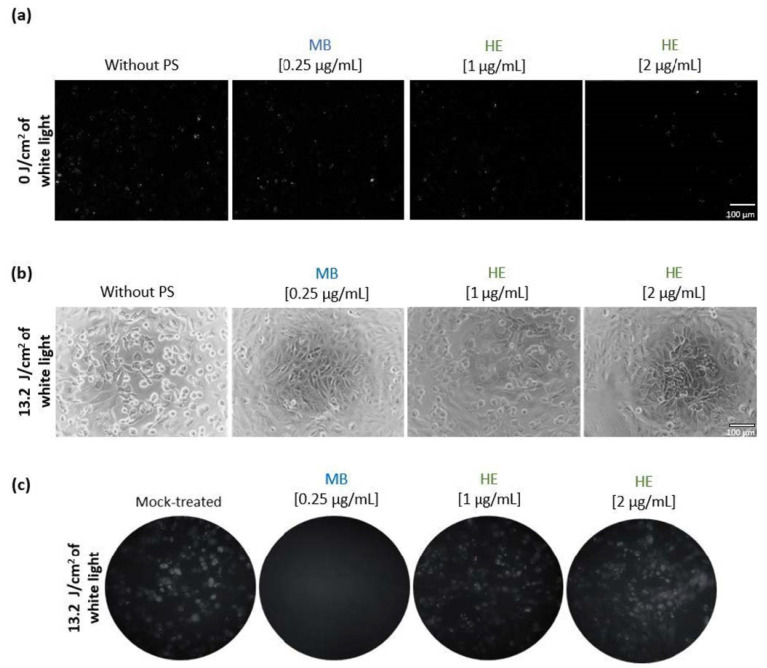
Effect of HE and MB in Vero cells alone or in combination with white light and infected with HSV-1 K26-GFP. Fluorescence (**a**,**c**) and brightfield images (**b**) of Vero cells monolayers infected with HSV-1 K26-GFP at an m.o.i. of 1. The virus was preincubated with 0.25 µg/mL of MB or 1–2 µg/mL of HE and irradiated or not with 13.2 J/cm^2^ of white light. Fluorescence microscopy images (**a**,**c**) show GFP signal corresponding to viral infection and brightfield images (**b**) report the CPE at 24 h p.i. Scale bars correspond to 100 μM in (**a**,**b**). Images in section (**c**) were taken at 10X focus.

To study the effect of these PSs on viral infection, Vero cells were cultured in 24-well tissue plates and infected or mock-infected with HSV-1 K26-GFP. Prior to infection, the virus was incubated with MB (0.25 µg/mL) or HE (1–2 µg/mL) in GM for 45 min. Then samples were infected with HSV-1 and some of them were irradiated with white light (13.2 J/cm^2^), and finally, all were incubated for 15 min at room temperature under dark conditions. The immunofluorescence images reported no significant difference in viral infection between Vero cells infected with the viruses treated or non-treated with the PS under dark conditions (Figure 2a). Nevertheless, a reduction in viral infection was observed when Vero cells were infected with irradiated MB-treated virus, and no CPE was observed during brightfield microscopy (Figure 2b) or the fluorescence images (Figure 2c). On the other hand, the virus incubated with irradiated HE showed no difference in infection compared to the irradiated sample without PS. In this case, the brightfield images show a remarkable CPE in the Vero cells infected with the virus previously treated with irradiated HE (Figure 2b). In addition, the GFP signal of HSV-1 K26-GFP was also detectable in the fluorescence images after irradiated HE-treated virus infection (Figure 2c).

### 3.3. Virucidal Effect of HE against HCoV-229E

**Figure 3:**
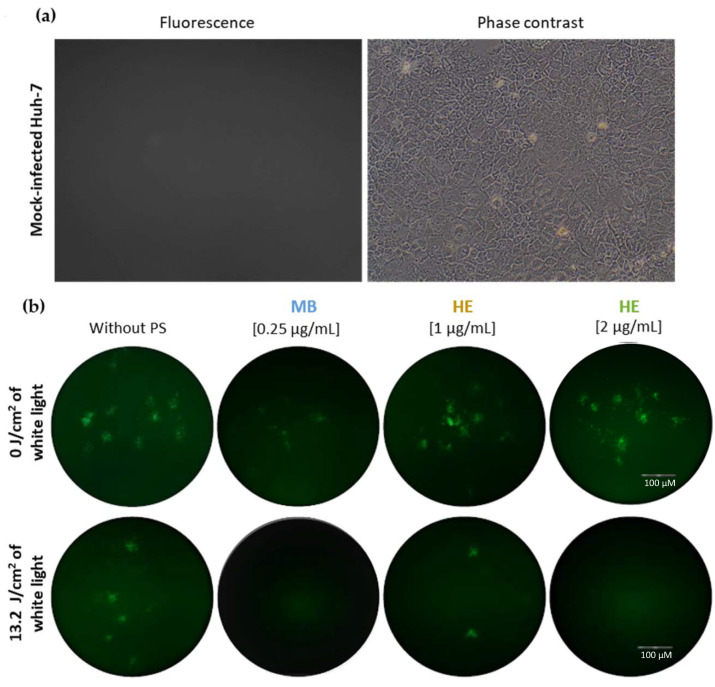
Effect of HE and MB in Huh-7 cells alone or in combination with white light and infected with HCoV-229E at an m.o.i of 0.5. (**a**) Fluorescence (left) and phase-contrast (right) images of Huh-7 cell morphology cultured in a monolayer and susceptible to HCoV-229E infection or mock of infection. Images were taken them at 20X. (**b**) Fluorescence images of the inhibition of HCoV-229E infection in Huh-7 cells induced by MB (0.25 µg/mL) and HE (1 µg/mL and 2 µg/mL) after irradiation or no irradiation with 13.2 J/cm^2^ of white light. HE and MB were incubated with the virus for 45 min. Subsequently, the samples were irradiated or non-irradiated and re-incubated for an additional 15 min. Immunofluorescence images were taken 48 h p.i. and GFP signal corresponds to infected cells. Images were taken at 10X focus. Scale bar corresponds to 100 μM.

The virucidal effect of HE against HCoV-229E was analyzed in the Huh-7 cell line. This cell culture grows in a monolayer, does not present an autofluorescence signal (Figure 3a) and is susceptible to infection by HCoV-229E. Huh-7 cells were cultured in 24-well plates and infected or mock-infected with HCoV-229E at an m.o.i of 0.5. Prior to infection, the virus was treated as described above. Fluorescence microscopy performed at 48 h p.i. did not report differences in the viral GFP signal in Huh-7 cells infected with HCoV-229E and previously treated with non-irradiated PSs, compared to untreated cells under dark conditions (Figure 3b). In contrast, a more significant decrease in infection was noted in cells when the virus was treated with irradiated HE. The infection was qualitatively decreased when the virus was treated with 1 µg/mL of irradiated HE, and it was considerably reduced with 2 µg/mL of irradiated HE -prior to infection, showing a dose-dependent virucidal effect (Figure 3b). HE presented a value of half the maximum inhibitory concentration (IC50) of 1.37 ± 0.2 µg/mL (Figure S4). The virucidal effect of MB against HCoV-229E was also corroborated, with an undetectable infection signal after the treatment of the virus with the irradiated compound (the brightfield is shown in Figure S3).

**Figure 4:**
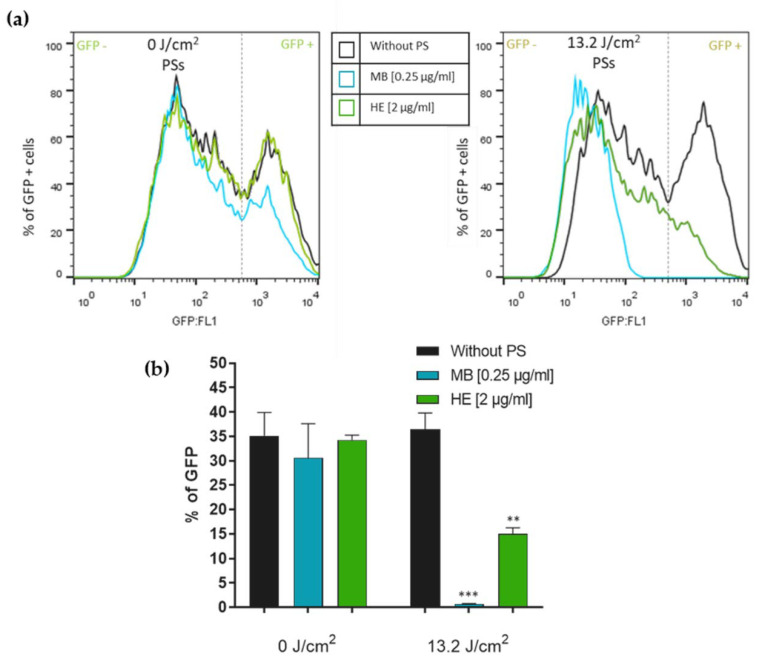
Inhibition of HCoV-229E infection (which expresses GFP) in Huh-7 cells induced by the PSs HE and MB and determined by flow cytometry. HCoV-229E was incubated with either MB (0.25 μg/mL) or HE (2 μg/mL) for 45 min. Subsequently, the samples were irradiated or not at 13.2 J/cm^2^ and re-incubated for an additional 15 min. Huh-7 cells were then infected with these samples at an m.o.i. of 0.5. Quantification of the infection was performed after 48 h p.i. by flow cytometry. (**a**) Plots obtained using FACScalibur Cytometer showed a decrease in the peak of GFP signal in Huh-7 cells after infection with irradiated PSs. (**b**) Bars show the percentage of GFP signal. Values are reported as the mean ± S.D. (*n* = 3; ** *p* < 0.005; *** *p* < 0.001).

The virucidal effect of HE against HCoV-229E was evaluated by flow cytometry. Huh-7 cells were cultured in a 24-well plate and infected or mock-infected with the treated virus with PSs, as described above. The results showed how the infection of cultures was significantly reduced after the virus was treated with the aPDT (1 h with the PSs in dark + 13.2 J/cm^2^ white light) (Figure 4a,b). Huh-7 cellular cultures infected with the virus without PS that were irradiated (13.2 J/cm^2^) or kept under dark conditions (0 J/cm^2^), revealed a percentage of GFP-HCoV-229E signal close to 37%. This percentage decreased to less than 1% in cells infected with irradiated MB-treated virus, and to 15% in cells infected with irradiated HE-treated virus.

**Figure 5:**
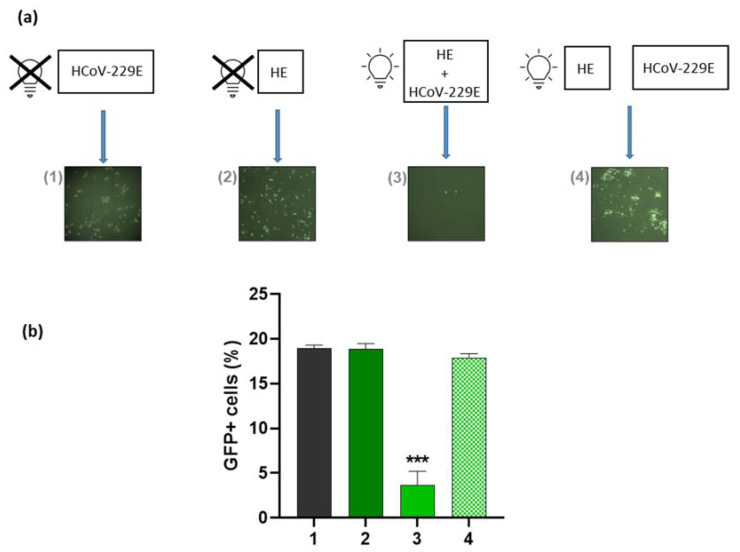
aPDT activity of HE in Huh-7 cells against HCoV-229E. Inhibition of HCoV-229E infection in Huh-7 cells induced by HE determined by fluorescent microscopy (**a**) and flow cytometry (**b**). Several conditions were assayed to demonstrate the virucidal activity of aPDT (HE + white light): (1) Infection with HCoV-229E; (2) HCoV-229E was incubated with HE during 1 h prior to infection; (3) HCoV-229E was incubated with HE for 45 min, irradiated with white light at 13.2 J/cm^2^ and re-incubated for an additional 15 min, prior to infection; (4) HE was irradiated with white light at 13.2 J/cm^2^ and added to HCoV-229E at the time of infection. For this experiment, pretreatment was performed with 2 µg/mL HE and virus at an m.o.i of 0.5. Huh-7 cells were infected with a 10-fold dilution of this mixture. In all conditions, cells were left in culture medium after 1 h of viral adsorption, washed with PBS and quantification of the infection was performed at 48 h p.i. by flow cytometry. Bars show the percentage of GFP signal. Values are reported as the mean ± S.D. (*n* = 3; *** *p* < 0.001). Images in (**a**) section were taken at 10X focus.

To confirm that the direct effect of HE as a PDT against HCoV-229E takes place at the time of irradiation, and in order to rule out any side effects at the time of pre-incubation, HE without virus was irradiated and then mixed with it in the cells. Infection was positive when the cells were infected only with the irradiated HE inoculum and the viruses were added, whereas cells infected with pre-irradiated HE and the virus together showed low infection rates (Figure 5a). The infection was quantified by flow cytometry, supporting the same results (Figure 5b).

**Figure 6:**
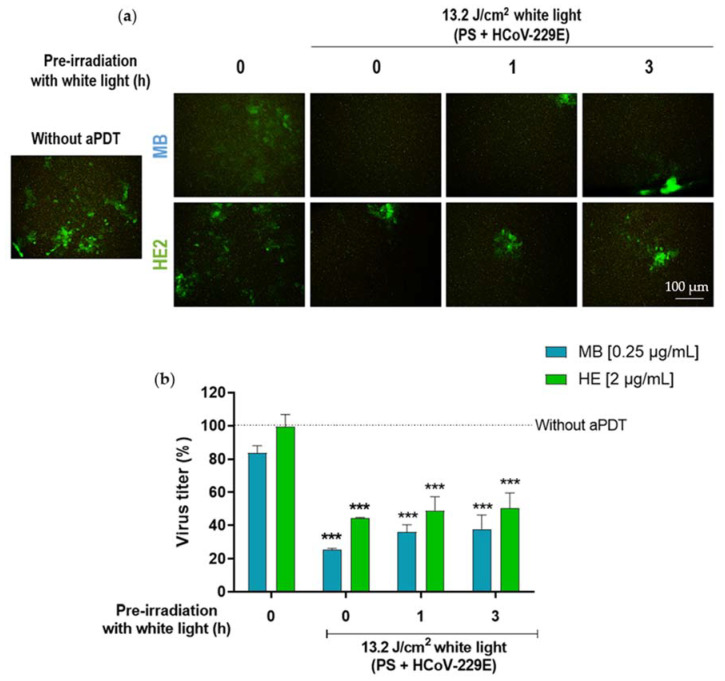
Evaluation of the photodegradation of HE and MB. (**a**) Fluorescence microscopy images of Huh-7 cells infected at an m.o.i of 0.5 with HCoV-229E (which expresses GFP) and treated with MB (0.25 µg/mL) or HE (2 µg/mL). (**b**) Virus titer plot compared positive control of infection without aPDT. The different conditions are: (I) positive control infection without aPDT; (II) PSs alone; (III) aPDT (1 h with PSs in dark + 13.2 J/cm^2^ white light); (IV) pre-irradiation of PSs for 1 h followed of aPDT; columns represent the mean titer ± S.D. (*n* = 3) (*** *p* < 0.001).

After the virucidal effect was demonstrated with the HE and white light, we wondered whether this PS had a high rate of photodegradation. To evaluate this, we pre-irradiated the PSs (0.25 µg/mL MB and 2 µg/mL HE) for 1 and 3 h with white light. Subsequently, we used the pre-irradiated PSs for aPDT against HCoV-229E (1 h with the PSs in dark + 13.2 J/cm^2^ white light). Figure 6a shows fluorescence microscopy images of the infectivity of HCoV-229E in Huh-7 cells (m.o.i. 0.5) under different conditions. The PSs alone did not induce significant differences with the mock infection, as in previous results. The aPDT (1 h with the PSs in dark + 13.2 J/cm^2^ white light) induced a significant decrease in the virus titer. These reductions were not modified when we used the pre-irradiated PSs (Figure 6a). This means that HE and MB remain effective as PSs after 1 and 3 h of white light irradiation (the brightfield microscopy images are shown in Figure S5).

## 4. Discussion

Despite the speed at which several vaccines against emergent SARS-CoV-2 have been approved, there are still no effective protective treatments against this virus. The development of new virucidal or antiviral treatments to combat HCoVs is a crucial strategy [35]. MB and TBO have been used in aPDT for quite a few years as a great option for antibiotic and antifungal treatments [18]. Moreover, aPDTs have been used to fight viral infections, as phenothiazines can photoinactivate viral particles via the oxidative damaging of their genetic material [36,37]. Their clinical application is mostly against skin, mucous and dental infections, due to the difficulty of applying the light in interstitial areas because of its limited penetration [38]. Here, we propose HE as a PS in combination with white light as a virucidal compound against HCoVs. HE is approved by the EMA for clinical uses [39], so its application for example as a hand disinfectant, would be very feasible and even more so in these times when the use of natural botanical extracts is so widespread [40]. However, hypericin alone is not yet approved by the EMA. Although it may be better to use hypericin as a PS, the fact that we have observed this potent virucidal activity with HE is very important. Therefore, we do not consider it essential for the purpose of this work to identify the components of the HE, other than knowing that it contains 0.3% hypericin, which is the percentage usually contained in the HE [29].

First, the MTT assay reported that HE and the aPDT (HE + with light) does not exert any cytotoxic effects in the cell lines tested. Therefore, we proceeded to evaluate the effectiveness of irradiated HE as aPDT against HSV-1 and HCoV-229E. The fluorescence assay of the Vero cells infected with HSV-1 performed in this study did not show any effect of HE against the virus. The opposite results were obtained by Spitzer et al., where a virucidal effect against HSV-1 was observed with treatments of HE ranging from 3.12 to 50 µg/mL [41]. Higher concentrations of HE and exposure to white light [42] could explain the disparity in the results obtained. The exposure of HE to white light did not produce any different results in cells infected at an m.o.i. of 1. Another study showed a reduction of HSV-1 infectivity from 0.5 log to 3.8 log in Vero cells at an m.o.i of 0.01 after the virus was in contact with HE [43]. This result indicates a possible virucidal effect of HE against HSV-1 when the virus dilution is 100-fold higher compared with our conditions of infection. In this study, cells infected at an m.o.i. of 1 with HSV-1—a standard concentration for viral assays—and pretreated with 1–2 µg/mL of HE showed a CPE and viral GFP-signal similar to infected cells without an aPDT ones. MB is one of the most widely used PSs against various infectious agents with a high rate of effectiveness even in the absence of light-induced activation [25,44]. However, we used a much lower concentration of MB compared to other studies describing its virucidal effect under dark conditions (3 to 0.25 ug/mL) to avoid this effect [45]. Consistent with previous studies [37,46], MB was used as a positive control of the virucidal effect of PDT. Parallel treatments with 0.25 μg/mL MB were carried out under the same conditions as the HE treatments. MB showed a virucidal effect against HSV-1 infection at an m.o.i of 1 on both non-irradiated and irradiated PS, being highly effective after the 13.2 J/cm^2^ of irradiation.

Otherwise, the infection caused by HCoV-229E was significantly reduced after the treatment of the virus with aPDT (HE + with light). The inhibition of the infection is HE-dose-dependent, evidencing the direct relationship between the irradiated HE and the inhibition of the virus. This effect is reproducible only after irradiation with white light. In fact, the lack of inhibition of HSV-1 and the necessary presence of light for an adequate virucidal function against this HCoV excludes the possible effect of HE on virus membrane destabilization [42,43]. The results obtained in this work are complementary to those obtained by Delcanale et al. [47], where the affinity of HE for the membrane of HCoV-22E is demonstrated and perhaps HE could play a possible role in it at higher concentrations or in different settings. Several studies of HE or Hypericin as a treatment against different HCoVs are focused on the disruption of the main proteinase (Mpro) function of the virus [48,49]. Mpro is an enzyme that plays an essential role in the first steps of infection on the translated viral RNA [50]. Previous in silico studies, where the potential interaction between the PS and Mpro was demonstrated [49,51], had been already confirmed in vitro, obtaining a decrease in viral infection [38,52].

Whereas other studies performed post-HCoVs-infection-treatment demonstrated an antiviral effect of hypericin targeting 3CL [52], where the compound is maintained throughout the infection, we propose a treatment prior to infection, using HE as a PS of aPDT, together with white light irradiation, as a virucidal compound. An early pretreatment with irradiated HE could present a complementary effect against the virus. In the presence of white light and oxygen, HE generates superoxide radicals that can derive into hydroxyl and peroxyl radicals, and single oxygen molecules which could block or inactivate viral particles [30,41,53] as is demonstrated in other studies. It has been observed how these superoxide radicals damage the genome of RNA viruses [23]. RNA viruses, such as HIV1, HIV2, and Dengue virus suffered hypericin photosensitive damage on their genome, disturbing the viral RNA and blocking or inhibiting viral growth and breeding [54,55]. It should be noted that one of the main advantages of using HE as a PS is that it is effective with the administration of white light, since daylight or sunlight is the main source of light outdoors [56]. Furthermore, we have demonstrated that even when HE is exposed to white light for 3 h, its phototoxic capacity is not inhibited, which shows that despite continuous exposure to white light, HE has long durability, being able to maintain the inhibition of viral infection.

## 5. Conclusions

In conclusion, the virucidal activity of HE in combination with white light, as aPDT against HCoV-229E infection in vitro it been demonstrated for the first time in this study. In contrast to an antiviral effect, this photosensitizing damage would affect the virus prior to its entry into cells. Our hypothesis of damage to the virus genome by the superoxide radicals is a feasible approach for future studies. In this regard, the use of photodynamic therapy with white light, to take advantage of sunlight, is a promising strategy for decreasing the virulence and pathogenicity of the virus. As we proposed in this research, future studies would be necessary to unravel the effect of superoxide radicals in the virus genome and to better understand the virucidal effect of HE.

## Data Availability

Not applicable.

## Supplementary Materials

The following supporting information can be downloaded at: https://www.mdpi.com/article/10.3390/pharmaceutics14112364/s1, Figure S1: Photoactivation effect of *Hypericum* Extract in viral infection. Experimental design; Figure S2: Experimental design of Figure 5; Figure S3: Figure 3 with brightfield; Figure S4: Maximum inhibitory concentration (IC50) of HE against HCoV-229E; Figure S5: Figure 5 with brightfield; Data sheet: Phytochemical characterization of HE.

## Abbreviations

aPDT: antimicrobial PDTs; CIV: cytomegalovirus; CPE: cytopathic effect; FBS: fetal bovine serum; GM: growth medium; HCoVs: human coronaviruses; HE: *Hypericum* extract; HIV: human immunodeficiency virus; h p.i: hours post infection; HPV: human papillomavirus; HSV-1: herpes simplex virus type 1; MB: methylene blue; MERS: Middle East respiratory syndrome; m.o.i: multiplicity of infection; PDT: photodynamic therapy; PS: photosensitizer; ROS: reactive oxygen species; SARS: severe acute respiratory syndrome: TBO: toluidine blue; TCID50: 50% tissue culture infectious dose.
