# Supplementary material for: Potent Virucidal Activity In Vitro of Photodynamic Therapy with Hypericum Extract as Photosensitizer and White Light against Human Coronavirus HCoV-229E
Source: Pharmaceutics. 2022 Nov 2;14(11):2364. doi: 10.3390/pharmaceutics14112364 (PMC9693429; doi:10.3390/pharmaceutics14112364)
Supplement: Supplementary file 1 [file pharmaceutics-14-02364-s001.zip › HYPERICUM EXTRACT - Translated to ENG.pdf]

FQS FARMAQUIMICA SUR | 20 AÑOS ANIVERSARIO

C/. Carlo Goldoni, 32 - Polig. Ind. Guadalhorce
Telfs.: 952 240 988 / 952 240 794 / 952 237 602
Fax: 952 242 585 - 29004 MÁLAGA
e-mail: farmaquimicasur@farmaquimicasur.com
www. farmaquimicasur.com

Productos Farmacéuticos y Químicos

PRODUCT SPECIFICATION

Product name: **HYPERICUM EXTRACT - DRY**

BATCH: 13651/6

EXPIRATION DATE: 04/2021

PRODUCTION BATCH: J-180413-HP

Characteristics

DESCRIPTION: Brown powder with characteristic taste and odor
SOLUBILITY: Moderately soluble
LATIN NAME: Hypericum perforatum L.
EXCIPIENTS: Maltodextrin < 10 %
SOLVENT EXTRACTION: Water-Ethanol
EXTRACTION RATIO: 8:1
USED PART: herb

Assays

Parameter	Specification	Result
**SIZE**	80 MESH	Conforming (0)
**RELATIVE DENSITY**	40-60 g/100ml	51.0g/100ml (0)
**TOTAL ASHES**	< =10 %	5.27% (0)
**LOSS ON DRYING (1g/105ºC/2h)**	<= 5 %	4.89% (0)
**UV TITLE**	>= 0,3 % HYPERCIN	0.35% (0)
**RATIO**	8:1	Conforming (0)
**RESIDUAL SOLVENTS**	Cumple Ph.Eur. 7.0	Conforming (0)
**HEAVY METALS**		
**As**	< 2 ppm	Conforming (0)
**Pb**	< 3 ppm	Conforming (0)
**Cd**	< 1 ppm	Conforming (0)
**Hg**	< 0.1 ppm	Conforming (0)
**PESTICIDES**	USP36-561	Conforming (0)
**AFLATOXIN B2-B1,B2,G1,G2**	< 2 ppb - < 4 ppb	Conforming (0)
**TOTAL AEROBES**	< 10000 ufc/g	Conforming (0)

TESTINGLaboratories :0 Data supplied by the manufacturer or authorized distributor
Signed: CARMEN TOROLLO
Date of issue: 26/03/2020 (dd/mm/yyyy)

Departamento Técnico
Luis Fernández/Carmen Torollo

FQS FARMAQUIMICA SUR | 20 AÑOS ANIVERSARIO
C/. Carlo Goldoni, 32 - Polig. Ind. Guadalhorce
Telfs.: 952 240 988 / 952 240 794 / 952 237 602
Fax: 952 242 585 - 29004 MÁLAGA
e-mail: farmaquimicasur@farmaquimicasur.com
www. farmaquimicasur.com

Productos Farmacéuticos y Químicos

PRODUCT SPECIFICATION

Product name: **HYPERICUM EXTRACT - DRY**

BATCH: 13651/6

EXPIRATION DATE: 04/2021

PRODUCTION BATCH: J-180413-HP

Parameter	Specification	Result
**MOULDS AND YEASTS**	< 1000 ufc/g	Conforming (0)
**P. AERUGINOSA**	ABSENCE	ABSENCE (0)
**E. COLI**	ABSENCE	ABSENCE (0)
**S. AUREUS**	ABSENCE	ABSENCE (0)
**SALMONELLA**	ABSENCE	ABSENCE (0)
**BENZOPYRENS**	=< 10 ppb GC/MS	Conforming (0)
**PAH'S**	=< 50 ppb	Conforming (0)
**IRRADIATION**	=< 700	Conforming (0)

REMARKS: Meets manufacturer specifications. Free product, GMO, Allergen, BSE/TSE free product.
CONSERVATION: Keep in a cool, dark place, away from light. Store at a temperature not exceeding 30°C.
RATING: SUITABLE

TESTINGLaboratories :0 Data supplied by the manufacturer or authorized distributor

Signed: CARMEN TOROLLO
Date of issue: 26/03/2020 (dd/mm/yyyy)

Departamento Técnico
Luis Fernández/Carmen Torollo
