# Supplementary material for: Potent Virucidal Activity In Vitro of Photodynamic Therapy with Hypericum Extract as Photosensitizer and White Light against Human Coronavirus HCoV-229E
Source: Pharmaceutics. 2022 Nov 2;14(11):2364. doi: 10.3390/pharmaceutics14112364 (PMC9693429; doi:10.3390/pharmaceutics14112364)
Supplement: Supplementary file 1 [file pharmaceutics-14-02364-s001.zip › Phytochemical characterization of HE.pdf]

FQS FARMAQUIMICA SUR | 20 AÑOS ANIVERSARIO
GOOD MANUFACTURING PRACTICE GMP QUALITY PRODUCT
C/. Carlo Goldoni, 32 - Polig. Ind. Guadalhorce
Telfs.: 952 240 988 / 952 240 794 / 952 237 602
Fax: 952 242 585 - 29004 MÁLAGA
e-mail: farmaquimicasur@farmaquimicasur.com
www. farmaquimicasur.com

BOLETÍN DE ANÁLISIS

PRODUCTO: **HIPERICO EXTO SECO**

LOTE: 13651/6

CAD: 04/2021

LOTE DE FABRICANTE: J-180413-HP

CARACTERÍSTICAS

**DESCRIPCIÓN:**	Polvo marrón de sabor y olor característico
**SOLUBILIDAD:**	Moderadamente soluble
**NOMBRE LATÍN**	Hypericum perforatum L.
**EXCIPIENTE**	Maltodextrina < 10 %
**SOLVENTE EXTRACCION**	Agua - Etanol
**RATIO EXTRACCION**	8:1
**PARTE USADA**	Hierba

ENSAYOS

PARÁMETRO	ESPECIFICACIÓN	RESULTADO
**TAMAÑO**	80 MESH	Conforme (0)
**DENSIDAD RELATIVA**	40-60 g/100ml	51,0 g/100ml (0)
**TOTAL CENIZAS**	< =10 %	5,27 % (0)
**PERDIDA POR DESECACION (1g/105ºC/2h)**	<= 5 %	4,89 % (0)
**TITULO UV**	>= 0,3 % HYPERCINA	0,35 % (0)
**RATIO**	8:1	Conforme (0)
**SOLVENTES RESIDUALES**	Cumple Ph.Eur. 7.0	Conforme (0)
**METALES PESADOS**		
**As**	< 2 ppm	Conforme (0)
**Pb**	< 3 ppm	Conforme (0)
**Cd**	< 1 ppm	Conforme (0)
**Hg**	< 0,1 ppm	Conforme (0)
**PESTICIDAS**	USP36-561	Conforme (0)
**AFLATOXINA B1- B1,B2,G1,G2**	< 2 ppb - < 4 ppb	Conforme (0)
**AEROBIOS TOALES**	< 10000 ufc/g	Conforme (0)

PRUEBALaboratorios :0 Datos suministrados por el fabricante o distribuidor autorizado

Firmado: CARMEN TOROLLO

Departamento Técnico
Luis Fernández/Carmen Torollo

Fecha de Expedición: 26/03/2020

FQS FARMAQUIMICA SUR | 20 AÑOS ANIVERSARIO
GMP GOOD MANUFACTURING PRACTICE QUALITY PRODUCT
C/. Carlo Goldoni, 32 - Polig. Ind. Guadalhorce
Telfs.: 952 240 988 / 952 240 794 / 952 237 602
Fax: 952 242 585 - 29004 MÁLAGA
e-mail: farmaquimicasur@farmaquimicasur.com
www. farmaquimicasur.com

BOLETÍN DE ANÁLISIS

PRODUCTO: **HIPERICO EXTO SECO**

LOTE: 13651/6

CAD: 04/2021

LOTE DE FABRICANTE: J-180413-HP

PARÁMETRO	ESPECIFICACIÓN	RESULTADO
**MOHOS Y LEVADURAS**	< 1000 ufc/g	Conforme (0)
**P. AUREGINOSA**	AUSENCIA	AUSENCIA (0)
**E. COLI**	AUSENCIA	AUSENCIA (0)
**S.AUREUS**	AUSENCIA	AUSENCIA (0)
**SALMONELLA**	AUSENCIA	AUSENCIA (0)
**BENZOPIRENOS**	=< 10 ppb GC/MS	Conforme (0)
**PAH´S**	=< 50 ppb	Conforme (0)
**IRRADIACIÓN**	=< 700	Conforme (0)

OBSERVACIONES: Cumple especificaciones fabricante. Producto libre OGM, Alérgenos, BSE/TSE

CONSERVACIÓN: Envase bien cerrado.Conservar en lugar fresco y oscuro, alejado de la luz. Conservar a temperatura no superior a 30ºC.

CALIFICACIÓN: APTO

PRUEBALaboratorios :0 Datos suministrados por el fabricante o distribuidor autorizado

Firmado: CARMEN TOROLLO

26/03/2020

Fecha de Expedición:

Departamento Técnico
Luis Fernández/Carmen Torollo
