# Supplementary material for: Potent Virucidal Activity In Vitro of Photodynamic Therapy with Hypericum Extract as Photosensitizer and White Light against Human Coronavirus HCoV-229E
Source: Pharmaceutics. 2022 Nov 2;14(11):2364. doi: 10.3390/pharmaceutics14112364 (PMC9693429; doi:10.3390/pharmaceutics14112364)
Supplement: Supplementary file 1 [file pharmaceutics-14-02364-s001.zip › supplementary figures.pdf]

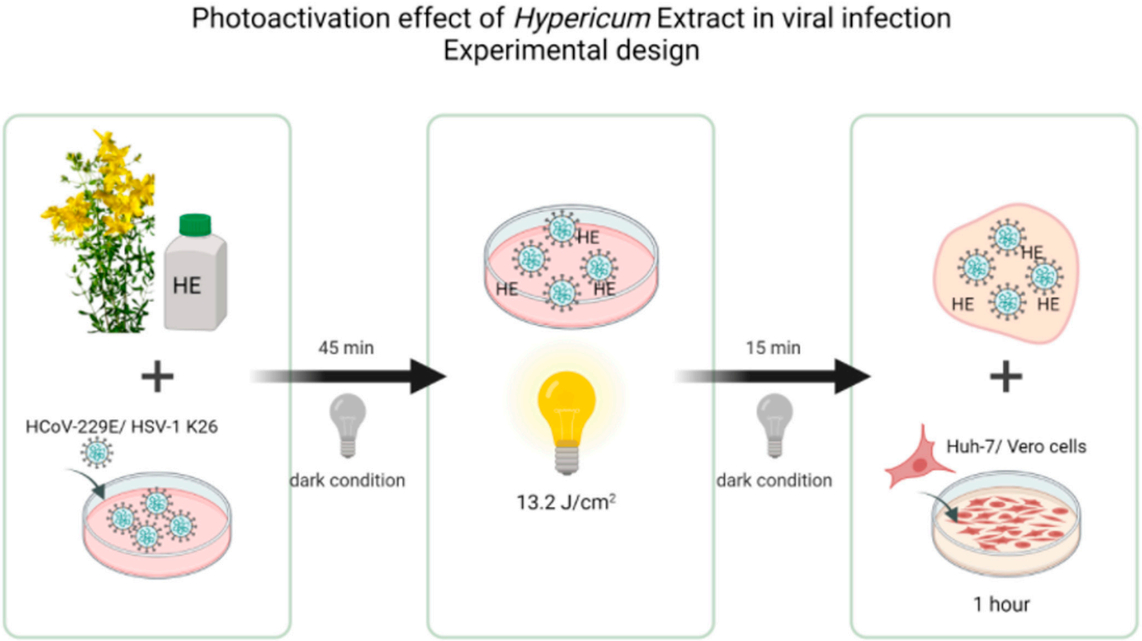

Figure S1. Photoactivation effect of Hypericum Extract in viral infection. Experimental design.

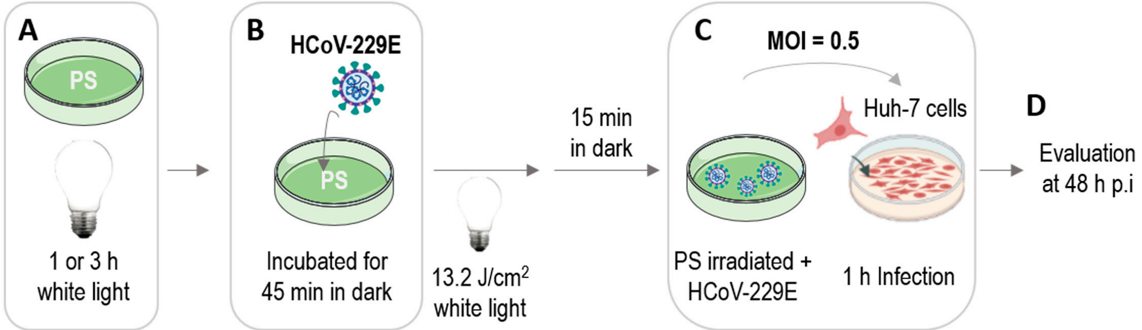

Figure S2. Experimental design of Figure 5.

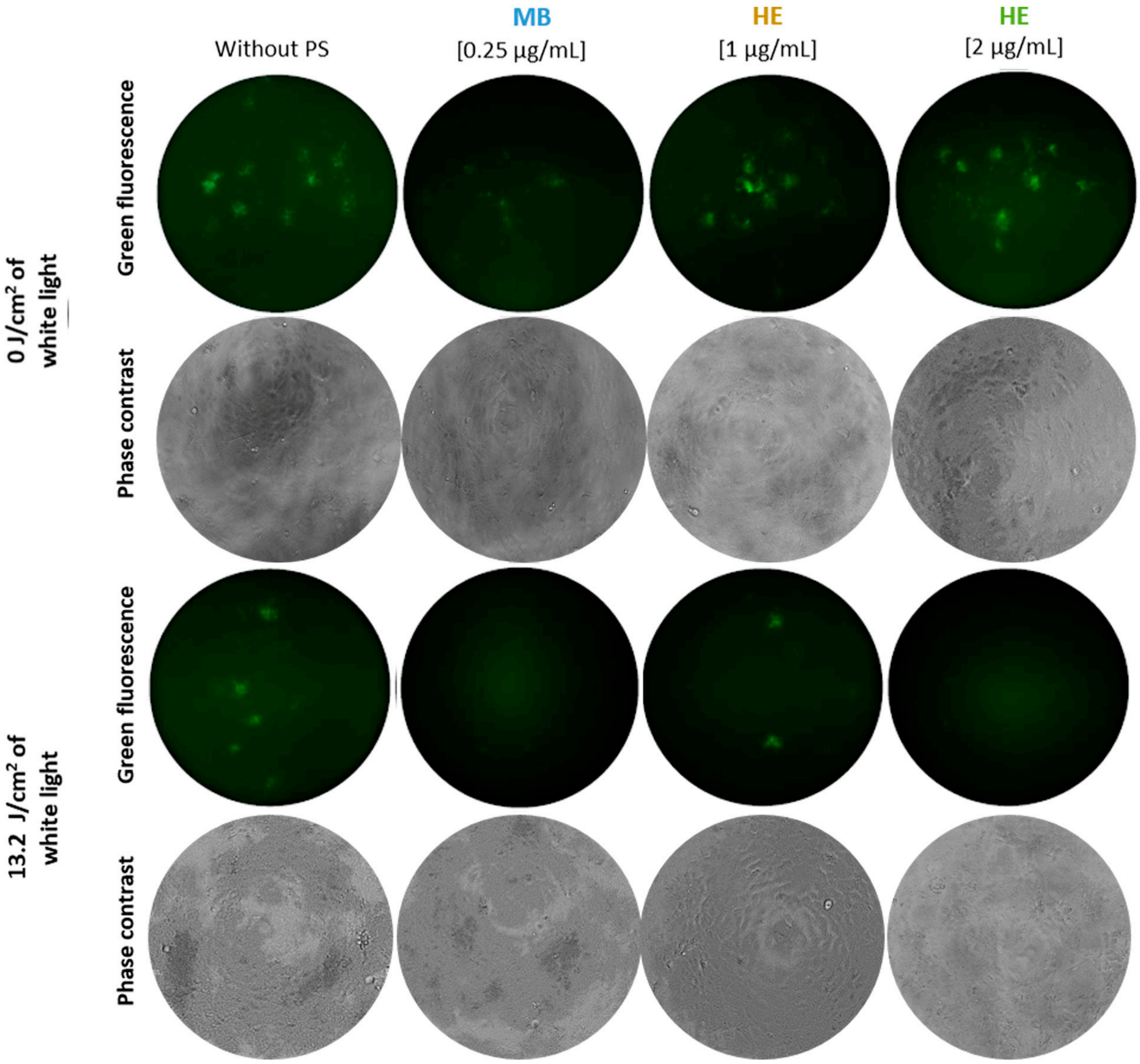

Figure S3. Figure 3 with brightfield.

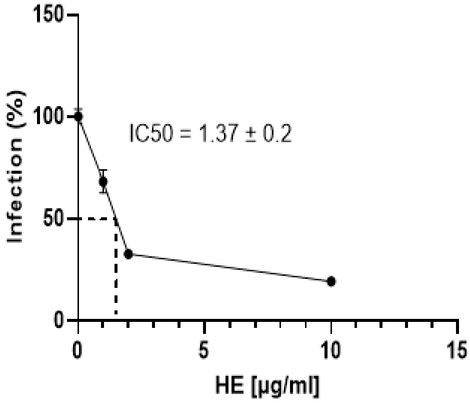

Figure S4. Maximum inhibitory concentration (IC50) of HE against HCoV-229E.

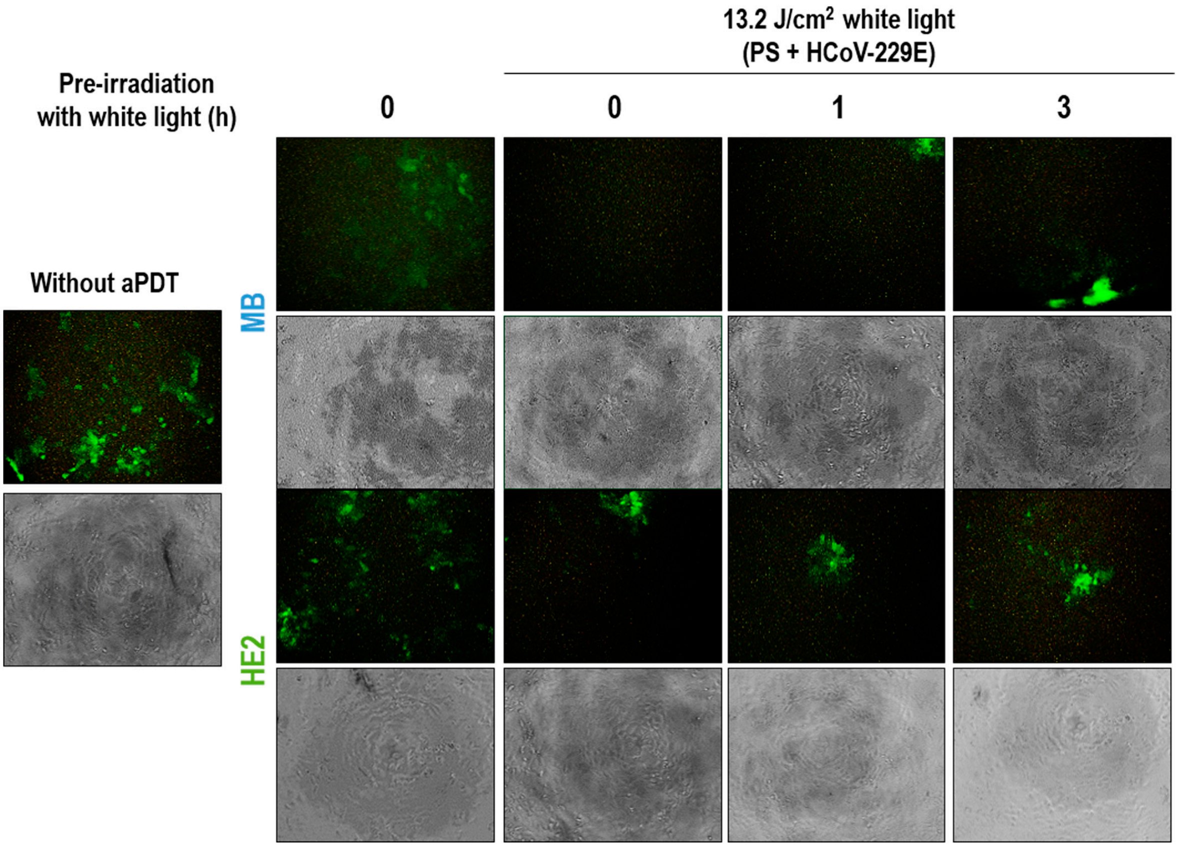

Figure S5. Figure 5 with brightfield.
